# Long-term Care Needs and Fuel Poverty among Older People: Beyond Energy Consumption and Affordability

**DOI:** 10.1007/s11205-025-03653-3

**Published:** 2025-07-08

**Authors:** Javiera Cartagena-Farias, Nicola Brimblecombe, Bo Hu, Sam Rickman

**Affiliations:** Care Policy and Evaluation Centre (CPEC), London School of Economics and Political Science, Houghton Street, London, WC2A 2AE UK

**Keywords:** Housing, Fuel poverty, Long-term care needs, Onset of care needs, Mental health

## Abstract

Both financial disadvantage and poor housing conditions are recognised as social determinants of poorer health and health inequalities across and within countries. In the UK and some other nations, low income and housing problems in interaction manifest as ‘fuel poverty’—a measure of a household’s ability to keep their homes warm. In these countries, tackling fuel poverty has become a priority, not least because of the known negative effects on health. Nevertheless, there is no gold standard measurement of fuel poverty, and there is also, more importantly, scant evidence on the relationship between fuel poverty and the development of long-term care needs among older people, which is important as understanding this relationship could inform preventative policy interventions aimed at reducing care needs and associated costs. Older people spend a larger proportion of their time indoors and the role of fuel poverty has wider health and social care impacts that go beyond immediate household hardship. This paper i) develops a data-driven measure of fuel poverty that goes beyond household income and energy consumption, ii) explores whether fuel poverty is associated with the development of care needs, an increase in care needs, and/or a decline in mental health among older people, and iii) whether there are any inequalities in the role played by fuel poverty across more potentially vulnerable groups. We use the English Longitudinal Study of Ageing, a large representative sample of people aged 50 or over. Structural Equation Modelling was used to provide a latent and comprehensive definition of fuel poverty. We found that fuel poverty is associated with a greater risk of developing long-term care needs and worse mental health. We also found that fuel poverty is multidimensional and as such, influences the development of care needs from many fronts. We provide evidence on the importance of reducing fuel poverty as a potential prevention mechanism of higher (or development of) care needs, and is particularly relevant in the current energy and cost-of-living crisis context in many countries.

## Introduction

Lack of quality housing, from poor sanitation to low insulation levels, has been identified worldwide as one of the key (avoidable) social determinants of health and health inequalities across and within countries (Marmot, 2005; World Health Organization, 2008). In many low-and middle-income nations, the focus has been on access to basic services, such as running water, or widening the coverage of electricity supply (United Nations, 2022). On the other hand, in high income countries, particularly in places with colder weather such as the UK, the emphasis has been given to housing conditions, including the difficulty to keep houses satisfactorily warm (Bouzarovski & Petrova, 2015; Preston et al., 2014). Cold housing conditions have been consistently linked to poorer well-being, physical and mental health outcomes (Churchill et al., 2020; Lloyd et al., 2008; World Health Organization WHO, 2018; Janssen et al., 2023; Public Health England, 2014; Marmot et al., 2010; Clair & Baker, 2022) and intrinsically related to the ability of a household to afford their energy bills, and therefore, to income and deprivation, a further determinant of ill health. Tackling fuel poverty has therefore become a priority in many developed countries, as it may reflect both household financial disadvantages and high fuel prices, as well as poorly insulated and energy-inefficient housing conditions. In the UK, for instance, the Warm Homes and Energy Conservation Act 2000 (HM Government, 2000) and the fuel poverty Strategy (Department of Trade and Industry, 2001) recognised fuel poverty as an important concern of public well-being, particularly among more vulnerable groups (Davillas et al., 2022; Sattari et al., 2023).

In this context, a growing amount of research has been providing evidence of the negative association between fuel poverty and health: Fuel poverty is linked with the development of respiratory illnesses and cardiovascular disease, as well as anxiety and depression in the UK (Marmot et al., 2010; Liddell & Morris, 2010; Davillas et al., 2022) and in other European countries (Heindl, 2015; Legendre et al., 2015; Llorca et al., 2020). However, there is scarce evidence on the potential relationship between fuel poverty and the development of long-term care (LTC) needs. Long-term care needs arise when an individual requires ongoing support in fulfilling activities of daily living, maintaining a living environment, developing personal relationships, or participating in social and community networks. Such needs can arise from declining physical or mental health, or cognitive impairment. It is unknown whether there is an association between fuel poverty and long-term care needs, particularly among older people and those living with long-term health problems, who have been identified as more at risk of suffering from cold housing conditions (Abdi et al., 2021; Chard & Walker, 2016; Public Health England, 2014; UK Health Security Agency, 2023). The latter may spend a large proportion of their time at home (Bouzarovski & Petrova, 2015). Cold indoor conditions have been linked to lower levels of blood circulation, increasing the risk of respiratory illness, such as asthma and pulmonary infections, and is associated with the constriction of vessels which provoke cardiovascular events, such as strokes, or even death (World Health Organization WHO, 2018). Nevertheless, the link between cold conditions and the development of care needs have not yet been established, nor explored in previous literature. We therefore sought to examine whether, controlling for physical health, fuel poverty is a predictor of developing long-term care needs. Our hypothesis being that colder indoor conditions may, among other consequences, reduce muscle strength, reduce balance, increase the risk of falls, and consequentially decrease independence levels (Janssen et al., 2023). Here it is important to notice that a third of older people fall at least one a year in England, with important consequences for individuals, but also for the health and care system (Office for Health Improvement & Disparities, 2022). Fuel poverty can be modified through public policy interventions, but the limited evidence about its relationship with the development of care needs makes it difficult to assess whether this is an effective strategy to reduce individual care needs or public costs.

Our study aims to explore the association between care needs and fuel poverty using a comprehensive concept of the latter. In this regard, we develop a data-driven, latent continuous measure of fuel poverty. This measure differs from the binary fuel poverty indicators, in favour of understanding fuel poverty as a situation in which a household finds it difficult to access services at the level required, therefore presenting a more comprehensive measure of a household’s ability to achieve and maintain indoor temperature at a comfortable level (Thomson & Snell, 2013). More specifically, this paper aims to i) develop a latent concept of fuel poverty including factors beyond household income and energy consumption, ii) explores whether fuel poverty is associated with the development of care needs, an increase in care needs, and/or a decline in mental health among older people, and iii) whether there are any inequalities in the role played by fuel poverty across potentially more vulnerable groups, such as those that live in social housing. The study investigates this issue in the English context, and it is relevant to policy makers at the local and national level, particularly in the current energy and cost-of-living crisis context, as well as in the likelihood of having more extreme weather in the future due to climate change.

### Measuring Fuel Poverty

Fuel poverty encapsulates a wide spectrum of circumstances (Boardman, 2012; Thomson et al., 2017; Hills, 2012), and therefore, there is no ‘gold standard’ measure for it (Deller et al., 2019). The need to develop policy strategies to tackle fuel poverty has led to governments in the UK to adopt fuel poverty binary indicators that are strongly based on the measurement of household income and energy needs, although lately also incorporate energy efficiency considerations. In this context, three different measures of fuel poverty have been implemented in the last decade in England, the FP10 indicator identifies households as living in fuel poverty if they spend more than ten percent of their household income on energy (Boardman, 1991; Davillas et al., 2022). Following consultations, the Low-Income-High Costs indicator (LIHC) was implemented in 2013 which records households as fuel poor if their required energy costs are above the national median and if their income minus energy costs falls below the income poverty line—which is sixty percent of the median national income (Hills, 2012). More recently, the Low Income Low Energy Efficiency (LILEE) indicator has been adopted. Under this new measure, a household is considered to be fuel poor if they are classified as being in fuel poverty under the LIHC indicator and if the property where they live has a low energy efficiency rating (Department for Business, Energy and Industrial Strategy, 2022). In 2010, 16.0 per cent of households were identified as living in fuel poverty under the FP10 indicator, in 2022, 13.4 per cent of households were living in fuel poverty in England under LILEE (Department for Business, Energy and Industrial Strategy, 2022; Department for Business, Energy and Industrial Strategy, 2012), but these figures are difficult to compare as they are a product of different definitions of fuel poverty.

The three definitions above include energy needs of households, which are, however, difficult to measure, and in practice they are being proxied by consumption and associated spending levels (Tu et al., 2022). Standard measures of fuel poverty may also mask some important factors. It has been suggested that more subjective indictors, including the perception of individuals on their ability to keep their house warm should also be included (Waddams Price et al., 2012). In addition, poor housing conditions may be considered, as they are understood as a catalyst for energy poverty when interacting with low income and energy prices (Deller, 2018). For instance, Oreszczyn et al. (2006) explored the effect of cold indoor environments in low-income households, finding that cold temperatures are associated with the energy performance of dwellings, the age of the property, and the average age and size of the household. Wider definitions of fuel poverty have also been proposed in the literature, e.g., the inability to access the energy levels needed to maintain physical, material, and social comfort (Bouzarovski & Petrova, 2015, pp.34). This includes not only the capacity of households to heat and cool their homes according to their needs, but also cooking, or being able to switch energy providers if desired.

For this study, we define fuel poverty in the spirit of a consensual poverty approach which understands poverty as the deprivation of all resources and experiences which are conventionally desired expected or prescribed in society (Veit-Wilson, 2009). We also incorporate aspects of the capability approach suggested by Sen (1993). The latter relates to the inability to achieve relevant and valued functionings, for instance, being in good health or being able to perform activities of daily living such as personal care. We therefore include in its estimation not only the information about energy consumption and household income, but also housing conditions reportedly associated with low efficiency levels, such as having issues with windows, roofs, or walls (Oreszczyn et al., 2006) and subjective measures of whether comfortable heating needs are met. For this, we estimate a continuous and latent measurement of fuel poverty, rather than a less nuanced binary indicator of fuel poverty. That is, we do not classify households as below or above an absolute or relative poverty line, but every household has an estimated level of fuel poverty given their characteristics and circumstances. Our approach is based on previous evidence findings, but it is also data driven, as the weighting of each factor associated with full poverty is determined by the data themselves and their pattern combinations.

### Fuel Poverty and an Ageing Population

The risk of falling into fuel poverty has been understood to be larger in the context of the energy crisis and more generally, the cost-of-living crisis which have been affecting many vulnerable households in England. Older people, in particular, may reside in older, less energy-efficient homes (Office for National Statistics (2022c). The latter may exacerbate health risks such as respiratory and cardiovascular conditions, which could lead to the loss of independence, and the need for support to perform daily activities, such as personal care and/or cooking. An ageing population may also reveal the consequences of living in fuel poverty over time, with increasing needs for services and associated costs to individuals, but also to the state. There is also great variability on how fuel poverty levels are distributed across the country. For instance, in 2013, the Department of Energy & Climate Change published fuel poverty estimations at small local-level-area. While some of them presented very low levels (2.0 per cent of households were identified as fuel poor), in others, 48.0 per cent of households were classified as fuel poor (Department of Energy & Climate Change, 2013).

## Methods

We use a large representative sample of the older population (and their partners) in England, the English Longitudinal Study of Ageing (ELSA). Four waves, waves 6 (2012/2013) to 9 (2018/2019), have been included in the analysis as they provide information on care needs of individuals and their mental health, as well as fuel poverty related information (Banks et al., 2021). ELSA also includes measures of local area deprivation rural–urban indicators (NatCen Social Research, 2021a, b), socio-demographic characteristics (such as education, ethnicity, gender, marital status, and employment status), housing conditions, and energy consumption. ELSA is a very rich source of information, and differs from the most common data collection used to explore fuel poverty in England (i.e. the English Housing Survey—EHS), because as a panel source of information, it allows the study of longitudinal trends and changes over time, as well as exploring the relationship between housing-related factors, income, energy consumption and needs, and the development of care needs. In addition, we have added local aggregated information from the domestic Energy Performance Certificates, EPC (Department for Levelling Up, Housing & Communities, 2022) to ELSA.

A total of 18,000 observations, from 4,701 individuals 50 years old or older, were available for analysis. Twenty seven percent of individuals lived in a household located in a rural area in wave 9, fifty-five percent of individuals in the sample were female in the same wave, the average age was 67 years old, and twelve percent of individuals lived in household located in areas identified as most deprived (i.e., in the fifth quintile most deprived index of multiple deprivation).

## Measures

### Fuel Poverty

Our latent concept of fuel poverty includes the following factors: Household income, energy (gas and electricity) expenditure, condensation problems, self-reported accommodation being too cold in winter, expectations of not having enough resources to cover needs, household location (rurality), and structural changes such as fuel prices at the national level. Weekly equivalised income is available for each individual in ELSA, taking into account differences in a household's size and composition by assigning different weights to adults and dependent children (UKDS, 2022). This approach is in line with the method used by the Government in its annual Households Below Average Income publication (Department for Work and Pensions, 2022). Weekly equivalised income has been multiplied by 4.34 to obtain monthly equivalised income and then multiplied by the number of adults living in the household to obtain the total monthly household income. Household energy requirements are not available in the dataset, but we have used as a proxy the total monthly amount in energy bills (gas and electricity) paid by the household. All methods of payment have been included (direct debit, pre-paid meter, bi-annual and quarterly bill). This proxy has the disadvantage that it may not necessarily reflect the necessary energy required to ensure thermal comfort (Tu et al., 2022). Therefore, we have also included information that better captures respondent perceptions of whether they are able to keep their house warm (‘whether their house is too cold in winter’, yes = 1; 0 = no) and their expectations with regards to not having enough financial ability to meet needs in the future (0 to 100%). Proxy measures of energy efficiency have also been included at the household level such as levels of condensation (yes = 1; no = 0), damp (yes = 1; no = 0), roof issues (yes = 1; no = 0), problems with windows (yes = 1; no = 0), and/or leaks (yes = 1; no = 0) as suggested by Deller (2018). We have also included the location of the household (rural = 1; urban = 0) as homes in rural areas have been, in general, found to be less energy efficient and usually rely on potentially more expensive heating fuels (Department for Environment, Food and Rural Affairs, 2022). Furthermore, we aimed to control by weather and structural factors to take into account, among other things, different calorific needs to keep household warm as well as difference in fuel prices over time. For this, we included a dummy variable for each wave of the ELSA data collection used in this investigation.

### Socio-demographic Characteristics

Our analysis also includes information on gender (female = 1; male = 0), ethnicity (White British = 1; non-White British = 0), highest level of education (university degree or above = 1; qualification below university degree or no qualification = 0), marital status (married or in partnership = 1, single = 0), and employment (employed = 1; not employed = 0). Local area deprivation levels (quintiles of the Index of Multiple Deprivation (NatCen Social Research, 2021a), 1 = least deprived to 5 = most deprived) were also included in the analysis, as a dummy variable that is equal to 1 if the household is located in an area in the two most deprived areas in the country and equal to 0 otherwise.

### Outcomes: Long-term Care Needs and Mental Health

Care needs have been measured as the total number of activities of daily living (ADLs) and instrumental activity of daily living (IADLs) for which individuals indicated that they need support. More specifically, the following question was asked to ELSA survey participants: “Please tell me if you have any difficulty with these activities because of a physical, mental, emotional or memory problem. Please exclude any difficulties you expect to last less than three months”. ADLs include activities such as bathing, eating, getting in and out of bed, and using the toilet. IADLs include recognising when in physical danger, preparing a hot meal, shopping for groceries, making telephone calls, taking medications, and managing money. Two main outcomes of interest were generated: i) the development of care needs (ADLs) for those without care needs in the previous period of analysis and ii) Changes in the total number of ADLs and IADLs over time.

Mental health was measured using the 10-item Centre for Epidemiologic Studies Depression Scale (CES-D) which has been widely used and validated in studies of late-life depression (Lewinsohn et al., 1997; Irwin et al., 1999). Unlike in the original CES-D Likert scale, in ELSA answers are given in yes = 1 and no = 0 format, so we added up the total number of answers positively indicating a mental health concern (we reversed some of the coding for this purpose). Thus, total scores range from 0 to 10, and high scores in the scale indicate a greater number of more severe depressive symptoms.

### Analysis

Our study explores the relationship between developing (or changes in) care needs and fuel poverty levels. It also investigates the association between the latter and mental health (number of depressive symptoms). For this, we use a comprehensive measure of fuel poverty that encapsulated a lack of affordability and thermal comfort (our latent, unobserved, variable). We perform Structural Equation Modelling (SEM). SEM represents the linear relationship between latent and observed variables, and is particularly valuable when studying latent concepts (i.e., those that are difficult to be directly observed or measured), or when observed variables are imperfect proxies of an umbrella concept (Chavance et al., 2010). In the case of fuel poverty, many factors contribute to the perceived inability of keeping warm, and how to combine them to determine who is classified as fuel poor, or not, has been a matter of debate for many years (Hills, 2012; Office for National Statistics, 2023a). Therefore, the use of SEM rises as a data-driven solution to the challenge of determining what is important when measuring fuel poverty and how to compose an indicator that consolidates the concept without the need to determine a priori how the relevant factors will be combined (as a standard composite measurement would do). This approach also allows the maintenance of the relevance and visibility of each relevant observed variable, rather than using a strategy to reduce dimensionality (such as the Principal Component Analysis).

The structural equation models included a ‘structural component’ where the latent concept of fuel poverty is defined based on relevant observed indicators and a ‘measurement component’ in which the relationship between fuel poverty and the relevant outcomes are defined. An important strength of treating fuel poverty as a latent construct in the measurement component of SEM is that it inherently accounts for measurement error in the observed indicators, resulting in a more reliable measure of fuel poverty. Latent orthogonality is assumed to ensure that each latent variable explains unique variance in the observed variables. In SEM, the coefficient of each path/arrow can be understood as the strength of the connection between the variables or outcomes analysed, keeping all other factors constant (Bollen, 1989; Knoke, 2005). We performed a SEM model for each outcome of interest: Changes in the level of care needs (using the total number of ADLs and IADLs in need of support), the development of care needs (ADLs), and mental health (depressive symptoms). In all these models, the continuous fuel poverty latent variable (common factor) was created based on household income, electricity and gas consumption, location (rurality), household expectation of their ability to cover their needs, perception of accommodation being too cold in winter, and rising damp (other housing problems associated with cold temperatures were also tested). Our models also consider the fact that the outcomes of interest are also driven by socio-demographic factors such as gender, ethnicity, education, employment, and marital status (the latter variable was only included in models exploring the relationship between fuel poverty and mental health). We have also accounted for physical health of individuals as this allows us to focus on care needs of individuals, and for structural changes over time, such as variability on fuel prices over the period of analysis. We also performed sub-sample analyses to investigate the possibility that older people living under different tenure arrangements could be substantially different, and as such the relationships between the age of development and fuel poverty could be different or have a different strength. Similarly, we explore the possibility that households located in low and high energy efficiency areas could present a different relationship. Low efficiency areas correspond to local authorities where more than 60% (median value) of their housing stock has an energy rating of D or below. On the other hand, high efficiency areas are local authorities with less than 61% of their housing stock has an energy rating of D or below. Energy ratings are based on data about the dwelling’s heating system and insulation level, among other things (Office for National Statistics, 2022a, b, c).

When performing SEMs, as the observed dimensions are available on different scales, standard deviations were used as measurement units for latent and observed variables (Chavance et al., 2010). To assess the overall fit of the model, we estimated several indices recommended to assess the fit of SEM models with continuous variables (Schreiber et al., 2008). The Root Mean Square Error of Approximation (RMSEA), the Comparative Fit Index (CFI), and the Non-Normed Fit (Tucker–Lewis) Index (TLI). The cut-off points used for a good model fit were < 0.06 for RMSEA, and > 0.95 for CFI and TLI indices as suggested by Hu and Bentler (1999). A cut-off RMSEA < 0.08 and TIL > 0.90 have been also suggested as reasonable model–data fit, but these thresholds are considered to be intuitive rather than statistical based (Marsh et al., 2004).

Finally, for comparative purposes, we explore the relationship between a standard measure of fuel poverty (e.g. FP10) and the development of care needs. We conducted analyses using Stata 17 (StataCorp, 2021).

## Results

In terms of the factors included in the latent (unobserved) concept of fuel poverty, as expected, higher household income is associated with a reduction in fuel poverty levels experienced by households. On the other hand, housing conditions, such as condensation, increase and also contribute to a higher level of fuel poverty. The subjective measure of feeling too cold in winter, as well as the expectations of not being able to cover needs in the future are associated with an increase in the level of fuel poverty in all the models analysed in Table 1. We also found that living in a rural area is negatively associated with the level fuel poverty, but these results were only statistically significant when changes in levels of care needs and mental health were analysed (please see Table 1). Models also controlled by each year/wave included in the analysis as part of the fuel poverty continuous latent concept. Here, we found that fuel poverty has been decreasing over time which is consistent with estimations using standard indicators (Office for National Statistics, 2022a), but this result needs to be taken with a degree of caution as data included in this analysis (and in official estimations) use pre-2021 figures, and therefore are not able to capture the current cost-of-living and energy crisis (with its associated high energy costs). Other potential factors associated with fuel poverty were also tested but dropped from the model due to convergence issues. For instance, housing problems with the roof, damp, and leakage were originally included.

**Table 1:** Structural equation model results

	SEM, Standardised Coefficient		
Standardised Coefficient	Probability of developing care needs (ADLs)	Changes in care needs (ADLs & IADLs)	Mental health (Depressive symptoms)
*Structural (t-1)*			
Fuel Poverty (latent variable)	0.676***	0.647***	0.409***
Care needs/Mental Health		−0.264***	0.500***
Female	−0.008	0.009***	0.056***
White	−0.005	−0.004***	−0.010***
Employed	−0.031**	0.006***	−0.029***
Higher education	−0.040***	−0.006***	−0.016***
Long-standing illness or disability	0.140***	0.139***	0.099***
Age	0.091***	0.124***	0.025***
Married/Partnership			−0.027***
Most Deprived quintiles (IV&V)	0.024***	0.012***	0.028***
_cons	−0523***	−0.932***	0.045***
*Fuel Poverty (t-1)*			
Gas and electricity monthly bill (£)	0.007	0.023***	0.025***
Household income (monthly)	−0.032*	−0.018***	−0.048***
Condensation	0.044**	0.017***	0.052***
Too cold in winter	0.002	0.033***	0.062***
Not enough £ to cover needs (expectations)	0.061***	0.017***	0.127***
Rural	−0.014	−0.024***	−0.006***
Wave 7 (ref. wave 6)	−0.016	−0.047***	−0.018***
Wave 8 (ref. wave 6)	−0.005	−2.92e-12***	−5.26e-6***
Wave 9 (ref. wave 6)	−0.010***	0.003***	0.016***
Number of observations	8,700	10,125	9,838
RMSEA	0.065	0.061	0.061
TLI	0.951	0.985	0.985
CFI	0.953	0.987	0.986

*p*-value: *** if < 0.001 (Significant at the 99% confidence level); ** if < 0.05 (Significant at the 95% confidence level); * if < 0.1 (Significant at the 90% confidence level)

The models fit were assessed by RMSEA (< 0.06), TLI (> 0.95), and CFI (> 0.95) goodness of fit measures

**Fig. 1:**
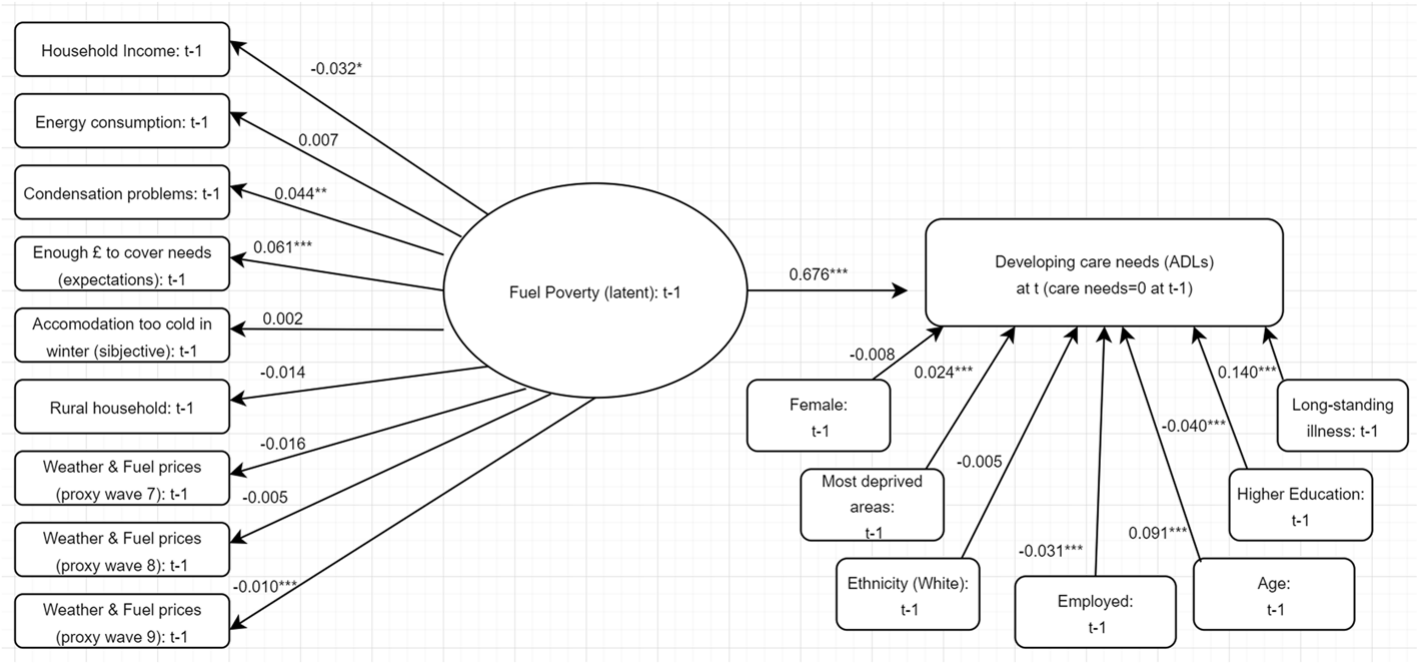
Structural Equation Modelling (SEM) results for the probability of developing care needs (ADLs), illustrative example

More specifically, we found that a higher level of fuel poverty, as a multi-dimensional concept, is associated with a significantly higher probability of developing care needs (ADLs) compared to those that do not live in fuel poverty by 0.676 standard deviations (RMSEA = 0.065; TLI = 0.951; and CFI = 0.953). Similarly, living in fuel poverty is associated with an increase in the number of ADLs and IADLs in need for support (by 0.647 standard deviations), and it was found to be associated with a deterioration of mental health, the latter measured using the depressive symptoms CES-D scale among older individuals (by 0.409 standard deviations). Fuel poverty itself was found to be associated with higher gas and electricity expenditure (other factors, including household income being kept constant). For instance, when looking at changes in care needs, an increase in 0.023 standard deviations in monthly bills is associated with an increase in one standard deviation in fuel poverty levels. Fuel poverty levels were also found to be negatively associated with monthly household income. Higher levels of fuel poverty experienced by households were also found to be associated with condensation, feeling too cold in winter, and the expectations of not being able to cover needs in the future. On the other hand, lower levels of fuel poverty were found to be associated with households located in a rural area. Table 1 presents the standardised coefficients (standard deviation units) for these three outcomes of interest. These results control for socio-demographics (gender, ethnicity, employment, education, age, and marital status) as well as whether or not the household is located in an area identified as being in the two most deprived quintiles in England. A representation of the relationship between the level of fuel poverty (and its constituent factors) and the development of care needs is found in Fig. 1 as an illustrative example.

Sub-sample analyses were performed for all the outcomes of interest, but we encountered convergence issues for the mental health and changes in care need outcomes. Table 2, therefore, shows the relationship only between fuel poverty and the development of care needs. This was done by type of housing tenure: social (public) rented, privately rented, and ownership. Here, the three sub-samples are large enough for a meaningful analysis, with the smallest group being privately renting, with 244 observations. As for the whole sample analysed, fuel poverty is associated with a greater risk of developing care needs across all types of housing tenure included in the analysis. However, with all other things being equal, the relationship is stronger for home owners (one more standard deviation in fuel poverty is associated with an increased risk of developing care needs of 0.677 standard deviation units) compared to those living in social housing (one more standard deviation in fuel poverty is associated with an increased risk of developing care needs of 0.645 standard deviation units) and those privately-renting (one more standard deviation in fuel poverty is associated with an increased risk of developing care needs of 0.660 standard deviation units). We also found inequalities in the factors contributing to the latent concept of fuel poverty. For instance, during the period of analysis, fuel poverty decreased over time, but this has benefitted homeowners and those privately renting more (0.023 and 0.025 standard deviation units decrease, respectively, at the last wave for a one-standard-deviation increase in fuel poverty) compared to those living in social housing (where we found 0.009 standard deviation units decrease at the last wave for a one-standard-deviation increase in fuel poverty). Condensation issues also has an unequal impact on those living in social housing and those privately renting (an increase in the risk of having condensation issues of 0.101 and 0.090 standard deviation units, respectively, for one standard deviation increase in fuel poverty) than on those living in their own homes (an increase in the risk of having condensation issues by 0.034 standard deviation units for one standard deviation increase in fuel poverty). There are also some differences in the strength of the relationship of fuel poverty and the expectations of not being able to meet needs in the future and finding their home too cold in winter. The latter two affecting those living in social housing the most.

**Table 2:** SEM, Standardised Coefficient: Development of care needs (ADLs) by housing tenure

	SEM, Standardised Coefficient: Development of care needs (ADLs)		
Standardised Coefficient	Social housing	Privately renting	Owner
*Structural (t-1)*			
Fuel Poverty (latent variable)	0.645***	0.660***	0.677***
Female	−0.048	−0.099	0.001
White	−0.045***	0.010	−0.005
Employed	−0.098***	0.010	0.004
Higher education	0.031	−0.089	−0.037***
Long-standing illness or disability	0.200***	0.047	0.139***
Age	0.091*	0.245***	0.117***
Most Deprived quintiles (IV&V)	−0.134	0.035	0.012
_cons	0.106	−1.252*	0.776***
*Fuel Poverty (t-1)*			
Gas and electricity monthly bill (£)	0.024	0.057	0.012
Household income (monthly)	−0.033	−0.03	−0.021
Condensation	0.101***	0.090*	0.034**
Too cold in winter	0.147**	0.061	0.018
Not enough £ to cover needs (expectations)	0.059***	0.056	0.040**
Rural area	−0.031***	0.053	−0.009
Wave 7 (ref. wave 6)	−0.005***	2.12E-09	−0.011
Wave 8 (ref. wave 6)	−0.004***	−0.088***	−0.060***
Wave 9 (ref. wave 6)	−0.009***	−0.025*	−0.023***
Number of observations	551	244	7,885
RMSEA	0.063	0.075	0.069
TLI	0.947	0.892	0.916
CFI	0.953	0.893	0.921

*p*-value: *** if < 0.001 (Significant at the 99% confidence level); ** if < 0.05 (Significant at the 95% confidence level); * if < 0.1 (Significant at the 90% confidence level)

The models fit were assessed by RMSEA (< 0.06), TLI (> 0.95), and CFI (> 0.95) goodness of fit measures

We also performed this analysis by splitting the sample across those living in a low and high energy efficiency area). Table 3 shows that while there is a larger association between fuel poverty on the development of care needs among those living in a low energy efficiency area (an increase of 0.674 standard deviation units) compared to those living in a high energy efficiency area (an increase of 0.628 standard deviation units). On the other hand, the negative association between household income and fuel poverty levels is strongly among those households living in more energy efficient areas (an increase in monthly income by 0.281 standard deviation units for one standard deviation increase in fuel poverty) compared to those living in less efficient energy areas (an increase in monthly income by 0.182 standard deviation units for one standard deviation increase in fuel poverty). This sheds light on how higher income levels could be dissipated due to higher energy needs associated with a lack of insulation.

**Table 3:** SEM, Standardised Coefficient: Development of care needs (ADLs) by energy efficiency area

	SEM, Standardised Coefficient: Development of care needs (ADLs)	
Standardised Coefficient	Low energy efficiency area	High energy efficiency area
*Structural (t-1)*		
Fuel Poverty (latent variable)	0.674***	0.628***
Female	−0.009	0.002
White	0.010	−0.036**
Employed	−0.004	−0.003
Higher education	−0.039***	−0.038**
Long-standing illness or disability	0.137***	0.143***
Age	0.122***	0.126***
Most Deprived quintiles (IV&V)	0.052	0.042**
_cons	−0.830***	−0.615***
*Fuel Poverty (t-1)*		
Gas and electricity monthly bill (£)	0.012	0.002
Household income (monthly)	−0.182**	−0.281***
Condensation	0.060***	−0.007
Too cold in winter	0.007	0.010
Not enough £ to cover needs (expectations)	0.076***	0.024
Rural area	−0.025	0.012
Wave 7 (ref. wave 6)	−0.033	−0.008
Wave 8 (ref. wave 6)	−0.029	0.007
Wave 9 (ref. wave 6)	−0.004***	−0.040***
Number of observations	5,177	3,523
RMSEA	0.069	0.073
TLI	0.952	0.922
CFI	0.961	0.941

*p*-value: *** if < 0.001 (Significant at the 99% confidence level); ** if < 0.05 (Significant at the 95% confidence level); * if < 0.1 (Significant at the 90% confidence level)

The models fit were assessed by RMSEA (< 0.06), TLI (> 0.95), and CFI (> 0.95) goodness of fit measures

For comparative purposes, when we performed a regression model to establish the relationship between the development of care needs and FP10, as well as mental health and FP10, we obtained similar results. Fuel poverty was statistically significantly associated to the outcomes of interest after controlling by individual-level characteristics (*p*-value = 0.027 and *p*-values = 0.001, respectively).

## Discussion

Our research is one of the first to investigate the association between fuel poverty and development of and changes in care needs, and it is novel in the use of a continuous latent concept of the former. We found that fuel poverty is associated with the development of care needs implying less disability free years for people living in fuel poverty, controlling for income, employment and socio-demographic factors. Higher levels of fuel poverty are also associated with increases in care needs, as represented by changes in number of ADLs and IADLs over time. Although there is no other research that we know of on fuel poverty and care needs, our findings are consistent with findings on the detrimental effect of fuel poverty on physical health (Marmot et al., 2010, 2020; Liddell & Morris, 2010; Davillas et al., 2022). We also found an association between fuel poverty and depressive symptoms, which is consistent with previous research findings, for instance, Davillas et al. (2022) found that the inability of households to keep their house warm is associated with worse mental outcomes.

Our analysis of the components of fuel poverty show that fuel poverty is associated to both individual factors such as household income, sense of financial security, electricity and gas consumption, and structural factors – poorer housing conditions—such as accommodation being too cold in winter and condensation issues. These factors are indicators of poor housing in themselves and also indicate energy inefficiency. The latent concept of fuel poverty developed in this study embraces other currently used measured of fuel poverty (e.g. FP10) as it also includes household income and energy costs, but it is more flexible in nature and data driven. Of the multiple aspects in our fuel poverty concept, both housing conditions (e.g. condensation, fuel prices) and material resources were associated with having care needs. This latter is consistent with other cross-sectional research showing that people on lower incomes have a higher prevalence of care needs (e.g. Brimblecombe & Burchardt, 2021). Including structural and individual determinants to the understanding of fuel poverty and its negative effects is important to fully understand who is most at risk and the policy and practice actions needed.

That the house a person lives in and their ability to keep it at an appropriate and comfortable temperature is related to development and level of care needs is a key inequality. There are wider equality ramifications. First, the association between fuel poverty and care needs were greater for some sub-groups of the population in our study. All else being equal (controlling for other factors), fuel poverty is associated with a lower risk of developing care needs among those living in social-rented housing compared to those privately renting or owner-occupied properties. In the UK, social housing properties are believed to have higher energy efficiency levels (Office for National Statistics, 2022c) meaning that some risk factors for fuel poverty are reduced. This finding is also consistent with the idea that many older households may be asset rich but cash poor, which perhaps keeps them from fixing their home (Hean et al., 2013; Fong et al., 2023). Second, fuel poverty is just one manifestation of socioeconomic deprivation. Those living in fuel poverty will experience general economic deprivation and may therefore be unable to cover other basic needs such as food.

We found that the fuel poverty decreased between 2012/2013 and 2018/2019, the period for which we had data available. This is consistent with other contemporaneous estimates of fuel poverty using other methods (Office for National Statistics, 2022a; Department for Business, Energy, and Industrial Strategy, 2019). This is a positive trend and reflect both energy prices and potentially efforts in some, albeit limited, areas to improve energy efficiency. However, that trend has not persisted and has reversed with rates of fuel poverty having increased since summer 2021 and predicted to increase further (Lee et al., 2022). Sharp increases in the costs of fuel have been a large contributor (Department for Energy Security and Net Zero & ONS, 2023; Office for National Statistics, 2023a). The rising cost of living (Harari et al., 2023) set against lower real-term household incomes especially for the poorest fifth of the population, and lower real-term welfare benefits may also have contributed (Office for National Statistics, 2023b). Regrettably, this has not been mitigated by actions to improve energy efficiency of housing, through for example better insulation. Whilst rates of installations to improve household energy efficiency in the UK increased up to 2012, there has been a drop by approximately 90 per cent since then (Lee et al., 2022). UK homes remain the least energy efficient in Europe. There is also widespread poor quality with 14% (3.4 million) of homes still failing to meet the basic minimum standards (Hodgkin & Sasse, 2022).

Action then is needed on fuel poverty to reduce inequality in care needs and improve health and quality of life. As an important component of fuel poverty is energy inefficient housing, which is also needed to mitigate the climate emergency. Predicted rises in fuel poverty and predicted extreme weather will exacerbate the situation, meaning the need for action is even more urgent. Fuel poverty comprises both individual determinants such as income and structural determinants such as housing condition and quality. Policy actions are therefore needed in both domains (Hinson et al., 2023). Income improvement measures could include adequate welfare benefit and employment income levels. It may also include dedicated help with the costs of heating the home. Recently, several countries have given help to households with fuel bills for example a cap on fuel costs (European Commission, 2021). The UK government makes payments to certain individuals to help with winter fuel costs (UK Government, 2023a, 2023b). However, whilst boosting income for the worse off has a wide range of benefits, increasing income to spend on fuel does not reduce carbon output. On the housing side, there are a number of potentially beneficial policy and practice actions. Stated intentions have been promising but have in large part come to little. A whole series of initiatives to advise on and advance energy efficiency have been under- or defunded, delayed, or have slowed down (Lee et al., 2022). There are implications of our findings for policymakers in countries where cold homes are an issue to both be more ambitious about fuel poverty strategy and for adequate, sustained and targeted funding of implementation. There are also implications for other countries. There are well-established links between fuel poverty and poorer health (Marmot et al., 2020; Davillas et al., 2022; Llorca et al., 2020; Heindl, 2015; Legendre et al., 2015), and between the components of fuel poverty – low income and poor housing conditions – and health (e.g. Marmot et al, 2010, 2020). We add to the picture, and reinforce the need for action but showing that these factors also contribute to earlier or greater care needs. Whilst fuel poverty may be a phenomenon of colder weather countries such as the UK, poverty and inadequate housing are not. Our findings therefore have international significance, and implications.

### Strengths and Limitations

The main strength of this study is that for first time (as far as we know) the concept of fuel poverty has been explored in association with the development of care needs among older people. Methodologically, we go beyond standard measures of fuel poverty that rely mostly on income and energy consumption, and created a latent (continuous) concept of fuel poverty that also includes housing conditions related to cold indoor temperatures (such as condensation) and a subjective measure of household’s ability to keep satisfactorily warm. We do, however, face some data limitations. We are assuming that care needs are reflected by ADLs and IADLs, but there may be unobserved factors among these traditional measures. Similarly, the size of the property (i.e. number of rooms) was excluded due to lack of completion. Energy efficiency scores were also used at local authority level, when ideally, we would have like to include them at individual household level. The latter was not possible due to the inability to obtain the exact location of households included in ELSA to protect their identity. We try to overcome this limitation by using a proxy measure such as housing conditions associated with insulation levels (such as condensation).

Multilevel Structural Equation Modelling (ML-SEM) to adjust by nested relationships were not considered in the analysis due to sample size limitations.

## Conclusions

This research provides timely evidence on the importance of reducing fuel poverty as a potential prevention mechanism for care needs or poorer mental health. There have been a range of international approaches to inflationary increases in fuel costs since the Covid-19 pandemic. In Scandinavia, Denmark, Norway and Finland introduced subsidies and tax credits to compensate households for rising energy prices (Greve et al., 2024). Conversely, the UK and Ireland, while introducing a general cap on charges per unit for households, have sought to reduce eligibility to fuel subsidies for older adults to households eligible for mean-tested benefits (Mackley et al., 2025; Kelly, 2025). The extent of the impact of such changes on fuel poverty is unknown and likely to be dynamic, particularly in the UK where the government has announced they wish to revisit this policy to widen eligibility (Hansard, 2025). Interventions which affect fuel poverty are a live policy issue. Policies that seek reductions in public spending, such as subsidies for winter fuel, may have unintended consequences and robust evidence on the consequences of fuel poverty – including effects on long-term care needs – is needed to inform decisions.

Using a comprehensive fuel poverty measure that incorporated individual and structural components, we found fuel poverty to be linked to an increase in the number of care needs, to the development of care needs, and to the number of depressive symptoms in older people. These associations are stronger for those living in owner occupied properties and those living in lower energy efficiency areas. Initiatives aimed at tackling all the components of fuel poverty, for example, boosts to income and reducing energy consumption via insulation, could protect households from its negative effects. Future research could be aimed at looking at the effect of fuel poverty on the use of health and social care services, and on the evaluation of strategies aimed to prevent household for living in cold homes.

Policy implications from this research are multiple, from the need to consistently improve insultation in house stocks in the country to providing vulnerable older people with the support they need to maintain comfort and suitability of their homes. Local and national strategies to prevent the development of care needs and the deterioration of physical and mental health are paramount to reduce pressure an associated cost to the health and care systems. In this regard, insulation programmes, interventions such as ‘Boiler on Prescription’ (Local Government Association, 2023), increasing regulation for private landlords, strategies to increase the ability of households to choose energy provider, and targeted welfare benefits support may prove to be beneficial.

## Data Availability

This study used third party data made available under licence that the author does not have permission to share (SN 5050 English Longitudinal Study of Ageing: Waves 0–9, 1998–2019; SN 8431 English Longitudinal Study of Ageing: Waves 1–8, 2002–2017: Quintile Index of Multiple Deprivation Score: Special Licence Access; and SN 8437 English Longitudinal Study of Ageing: Waves 6–8, 2012–2017: Census 2011 Rural–Urban Indicators (Recoded): Special Licence Access). Requests to access the data should be directed to the UK Data Service (UKDS) at https://ukdataservice.ac.uk/ Domestic Energy Performance Certificates (EPC) data are published by the Department for Levelling Up, Housing & Communities at https://epc.opendatacommunities.org/
